# Synthesis of Rice Husk-Based MCM-41 for Removal of Aflatoxin B_1_ from Peanut Oil

**DOI:** 10.3390/toxins14020087

**Published:** 2022-01-23

**Authors:** Ya’nan Li, Ren Wang, Xiaohu Luo, Zhengxing Chen, Li Wang, Yunyu Zhou, Weizhi Liu, Miaomiao Cheng, Chen Zhang

**Affiliations:** 1 Key Laboratory of Carbohydrate Chemistry and Biotechnology, Ministry of Education, Jiangnan University, Wuxi 214122, China; 89494900013@jiangnan.edu.cn (Y.L.); zxchen@jiangnan.edu.cn (Z.C.); 2 National Engineering Research Center of Cereal Fermentation and Food Biomanufacturing, Jiangnan University, Wuxi 214122, China; nedved_wr@jiangnan.edu.cn (R.W.); xh06326@jiangnan.edu.cn (X.L.); 6200113057@stu.jiangnan.edu.cn (W.L.); 6200112015@stu.jiangnan.edu.cn (M.C.); 3 Jiangsu Provincial Engineering Research Center for Bioactive Product Processing, Jiangnan University, Wuxi 214122, China; 4 School of Food Science and Technology, Jiangnan University, Wuxi 214122, China; wangli@jiangnan.edu.cn; 5 Wuxi Zodolabs Biotech Co., Ltd., Wuxi 214174, China; 6 Wuxi Xinwu Environmental Protection Technology Co., Ltd., Wuxi 214028, China; zcwwt@163.com

**Keywords:** rice husk-based MCM-41, aflatoxin B_1_, adsorption, peanut oil, economic value

## Abstract

Edible oils, especially peanut oil, usually contain aflatoxin B_1_ (AFB_1_) at extremely high concentrations. This study focused on the synthesis of rice husk-based mesoporous silica (MCM-41) for the removal of AFB_1_ from peanut oil. MCM-41 was characterized by X-ray diffraction, N_2_ physisorption, and transmission electron microscope. MCM-41 was shown to have ordered channels with high specific surface area (1246 m^2^/g), pore volume (1.75 cm^3^/g), and pore diameter (3.11 nm). Under the optimal concentration of 1.0 mg/mL of the adsorbent dose, the adsorption behavior of MCM-41, natural montmorillonite (MONT), and commercial activated carbon (CA) for AFB_1_ were compared. The adsorption of AFB_1_ in peanut oil onto the three adsorbents was slower compared to that of AFB_1_ in an aqueous solution. In addition, the pseudo-second-order kinetic model better fit the adsorption kinetics of AFB_1,_ while the adsorption mechanism followed the Langmuir adsorption isotherm on the three adsorbents. The calculated maximum adsorbed amounts of AFB_1_ on MONT, MCM-41, and CA were 199.41, 215.93, and 248.93 ng/mg, respectively. These results suggested that MCM-41 without modification could meet market demand and could be considered a good candidate for the removal of AFB_1_ from peanut oil. This study provides insights that could prove to be of economic and practical value.

## 1. Introduction

Aflatoxin B_1_ (AFB_1_), which contaminated peanut, corn, sorghum, oilseed, animal feed, and foods, was a secondary metabolite produced by *Aspergillus flavus* and *Aspergillus parasiticus.* It was ubiquitous in the fields and was able to grow on grains during the storage period [1]. The International Agency for Research on Cancer (IARC) listed AFB_1_ as one of the strongest carcinogens due to its high toxicity, teratogenicity, carcinogenicity, and mutagenicity. AFB_1_ at concentrations of 5–30% has been identified in raw peanuts and peanut-based products in the main peanut-producing regions in China [2]. Since peanut oil was commonly used as an edible oil in China, the contamination of peanut oil with AFB_1_ represents a serious public health issue. A previous study described high concentrations of AFB_1_ (38.74 ± 47.45 μg/kg) in home-cooked foods in China where peanut oil was used [3].

To reduce the risk of AFB_1_ contamination, several approaches for the detoxification of peanut oil have been proposed, including chemical (alkali [4] and ozone [5] treatments), biological (microbial adsorption and degradation [6]), and physical methods (UV irradiation [7,8], photocatalysis [9], and adsorption [10]). Although the detoxification rate of peanut oil could be around 99% when using UV irradiation, photocatalysis, or ozone treatment, new toxins with equal or high toxicity compared to AFB_1_ may be generated during the detoxification of peanut oil. Microbial degradation of AFB_1_ may be of limited application due to the reversibility of microbial adsorption [6]. Alkali treatment and adsorption were the most commonly used methods for the detoxification of peanut oil. However, chemicals generated during the alkali treatment of peanut oil could potentially be considered pollutants. The adsorption method was easy to perform, especially for oil substrates that cannot be detoxified by alkali treatment, such as peanut oil, sesame oil, and rapeseed oil. Therefore, it was proposed that the adsorption method may be an adequate strategy for the detoxification of vegetable oils contaminated by AFB_1_ [10]. To date, many types of adsorbents have been described as having the ability to adsorb AFB_1_, such as clay minerals [11,12] and organic/biological adsorbents [13,14]. Common mineral adsorbents include activated carbon, diatomite, attapulgite, and montmorillonite (MONT). MONT has been mainly investigated for its high adsorption capacity for AFB_1_. Furthermore, toxicological studies showed that the addition of MONT to human diets neither induced toxic effects [15] nor disrupted the equilibrium of minerals and vitamins in the blood, which reinforced the potential of using mineral adsorbents for the removal of AFB_1_ from peanut oil.

Mesoporous silica in the form of MCM-41 has attracted considerable attention for its high surface area, ordered porosity, narrow pore size distribution, easy regeneration, high thermal stability, and reusability. MCM-41 has been used in many applications in the fields of catalysis, adsorption, separation, chromatography, and others. Moreover, MCM-41 and its modified products exhibited a broad-spectrum capacity to adsorb many types of adsorbates, such as metal ions, dyes, gases, and drugs [16,17,18,19]. In addition, as-synthesized and modified MCM-41 exhibited good adsorption performance for organic compounds, particularly aromatic compounds such as benzene [20], mesitylene [21], phenol [22], toluene [23], aniline [24], and chlorophenol [25]. However, the chemical modification of MCM-41, which included amino, sulfhydryl, and metal modification, greatly increased synthesis costs due to the use of expensive reagents and complex modification methods, which limited the application of MCM-41 [26,27,28].

Tetraethyl orthosilicate (TEOS) was commonly used as a silicon source to synthesize mesoporous materials. However, TEOS was expensive, and the identification of cost-effective and alternative silica sources was in place. Rice was one of the most important food crops in China [29]. Therefore, rice husk was an extremely abundant material that was not fully utilized. It was thoroughly discarded or burnt in cultivated lands, thus causing considerable environmental pollution. Rice husk had approximately 20% of ash content in which the main component was amorphous SiO_2_ (>98%), which could be an appropriate silicon source [30]. MCM-41 synthesized using rice-husk ash has been used for the removal of toxic metals [31], dyes [32], pesticides [33], and pharmaceuticals [34], among other materials. It can be hypothesized that MCM-41 could be an excellent adsorbent for complex organic compounds such as AFB_1_. However, the application of rice husk-based MCM-41 on the removal of AFB_1_ from peanut oil has been rarely attempted [35].

Therefore, in this study, we aimed to investigate the mechanism and performance of rice husk-based MCM-41 in the detoxification of peanut oil from AFB_1_. MCM-41 was synthesized from rice husk and characterized in its structural, textural, and morphological aspects by X-ray diffraction (XRD), N_2_ physisorption, and transmission electron microscope (TEM), respectively. Moreover, the adsorption behavior for AFB_1_ of rice husk-based MCM-41 was compared with that of natural montmorillonite (MONT) and commercial activated carbon (CA). This study provided insights into novel ways to use rice husk to improve food safety. It also proposed the basis for a convenient detoxification technology with potential application in the grain and oil processing industry.

## 2. Results and Discussion

### 2.1. Characterization of MCM-41

**Figure 1:**
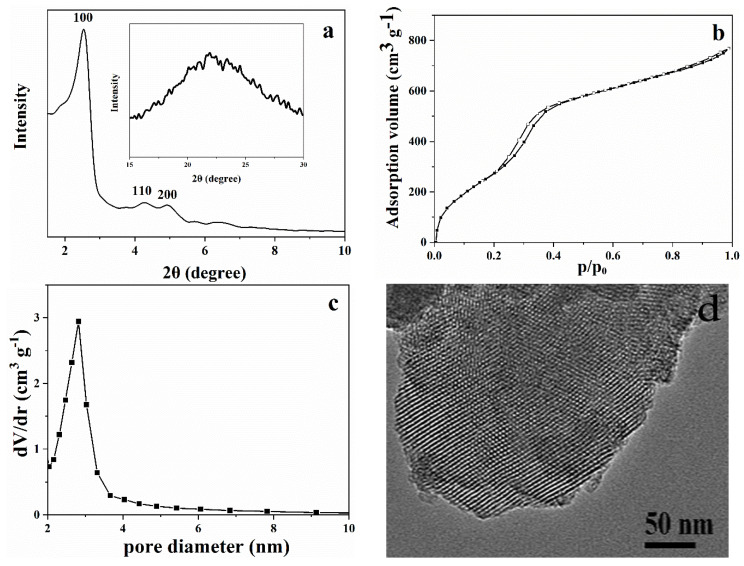
Characterization of MCM-41. (**a**) XRD patterns, (**b**) nitrogen adsorption–desorption isotherms, (**c**) pore size distributions, and (**d**) TEM image of MCM-41.

The XRD pattern of MCM-41 is shown in Figure 1a. The diffraction peak at 2θ of 2.56° indicated the (100) plane while the diffraction peaks at 2θ of 4.30° and 4.94° revealed the (110) and (200) planes, respectively. The diffraction peak of the (100) plane was evident and narrow, indicating that the synthesized MCM-41 had a hexagonal crystal structure with a high degree of long-range ordering, corroborating previously reported observations [36]. A diffuse peak at 2θ of 15–30° (Figure 1a) was indicative of the amorphous nature of the pore wall [29]. The nitrogen adsorption–desorption isotherm of MCM-41 was shown in Figure 1b. MCM-41 exhibited a type-IV isotherm of the IUPAC (International Union of Pure and Applied Chemistry) [35]. The adsorption–desorption isotherm showed a similar hysteresis loop in the pre-capillary condensation range (P/P0 = 0.2–0.4). This could be attributed to the phase transition of nitrogen adsorbent from a disordered low-density liquid state to an ordered, solid-like high-density state [37]. The tensile strength of the adsorbent increased with the increase in density and tension, which resulted in a sudden increase in adsorption capacity at P/P0 = 0.2–0.4, as suggested by Gregg and Sing [38]. No capillary condensation hysteresis loop at P/P0 = 0.4–0.8 was observed, which was in agreement with the findings of Thommes [39]. The width of the adsorption hysteresis loop was reduced with the decrease in the pore diameter, and the hysteresis loop was not observed at a pore diameter lower than 3.3 nm. The pore diameter of MCM-41 observed in this study was 3.11 nm (Figure 1c, Table 1). Surface area and pore parameters of MCM-41, MONT, and CA were provided in Table 1. MCM-41 displayed the largest specific surface area (1246 m^2^/g), which was in agreement with the structural features of mesoporous materials. These were characterized by a high specific surface area [40,41]. The specific surface area of MCM-41 was larger than that reported by Abbas [42] and Artkla [43]. Moreover, MCM-41 had higher surface area, pore volume, and diameter when compared with CA and MONT (Table 1), which suggested a promising adsorption capacity. TEM analysis of MCM-41 (Figure 1d) showed a highly ordered hexagonal array of mesoporous silica.

Collectively, these results confirmed the successful synthesis of MCM-41 mesoporous silica with uniform and ordered channels. This material was subsequently evaluated for its adoption capacity for the removal of AFB_1_ from peanut oil.

### 2.2. Effect of Adsorbent Dose

**Figure 2:**
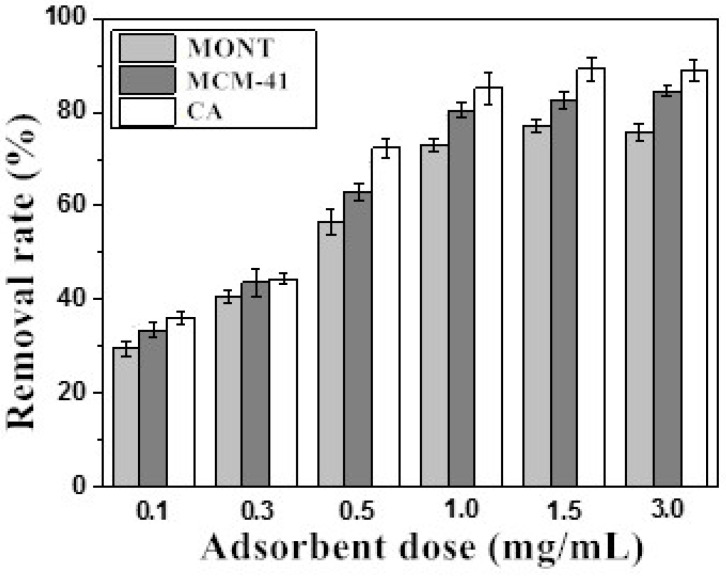
Removal rate of AFB_1_ from peanut oil considering the performance of three adsorbents (MONT, MCM-41, and CA) at different concentrations (0.1, 0.3, 0.5, 1.0, 1.5, and 3.0 mg/mL).

Adsorption of AFB_1_ by three adsorbents was carried out at 25 °C for 24 h. Figure 2 showed the removal rate of AFB_1_ by MONT, MCM-41, and CA against the adsorbent dose (0.1, 0.3, 0.5, 1.0, 1.5, and 3.0 mg/mL). Regardless of the adsorbent dose, the removal rate was higher when using CA, followed by MCM-41 and MONT. As expected, the removal rate of AFB_1_ at the adsorbent dose of 0.3 mg/mL was very low. The removal rate of AFB_1_ increased as the adsorbent dose increased, which could be explained by increased interactions between AFB_1_ and absorbents, including hydrogen bonding, pore filling, and electrostatic attractions. With an adsorbent dose of 1.0 mg/mL, the removal rates of AFB_1_ were 73.23%, 80.35%, and 85.09% when using MONT, MCM-41, and CA, respectively. At a higher adsorbent dose (1.5 mg/mL), the removal rates of AFB_1_ slightly increased to 75.56%, 83.48%, and 89.15% when using MONT, MCM-41, and CA, respectively. In this adsorption system, when adsorbents were used at lower concentrations, fewer adsorption sites were available; therefore, increasing adsorbent concentration effectively increased the number of adsorption sites and consequently improved the removal rate of AFB_1_. However, at excessively high concentrations, the phenomena of cementation, flocculation, and agglomeration among the adsorbent’s particles occurred. This led to a stabilization or a decrease in the number of available adsorption sites, and the removal rate did not improve further [44]. Moreover, micronutrients such as vitamin E and sterols might be eliminated when absorbents were employed at high concentrations, leading to nutrient loss [10]. Therefore, the use of adsorbents at a concentration of 1.0 mg/mL was considered optimal and was thus adopted in further experiments. In addition, MCM-41 at 1.0 mg/mL showed a good adsorption performance in the removal of AFB_1_ at a rate of 80.35%, which was similar to the results of Ji and Xie [10].

### 2.3. Adsorption Kinetics

**Figure 3:**
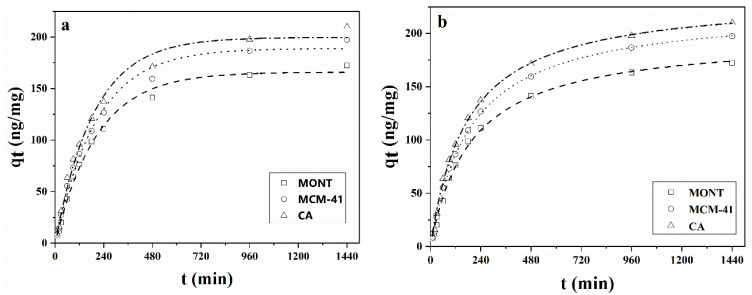
(**a**) Pseudo-first-order kinetic model fitting curves and (**b**) pseudo-second-order kinetic model fitting curves of AFB_1_ adsorption on MONT, MCM-41, and CA.

The efficiency of AFB_1_ adsorption by adsorbents evaluated in the present study was determined by adsorption kinetics in which the removal rate and adsorption time of AFB_1_ were measured. Adsorption kinetics of AFB_1_ by three adsorbents was performed at 25 °C with prolonged adsorption time. As shown in Figure 3a,b, rapid adsorption was observed in the initial 120 min, and no significant change in adsorption was verified after 960 min of treatment. This indicated that the removal rate reached an apparent equilibrium at this stage. The initial removal rate occurred relatively quickly since more adsorption sites located on the external surface of the adsorbent were available for reaction. However, as the external surface of the adsorbent was covered by AFB_1_ molecules, the adsorption rate was gradually reduced and then stabilized, suggesting that the adsorption of AFB_1_ on the inner surface of adsorbents occurred by interlayer diffusion [45]. Compared with previously published results [46,47], the slower adsorption kinetics of MONT, MCM-41, and CA in the oil phase compared to those in the aqueous solution could be due to the high viscosity of peanut oil (10.0 cP at 25 °C), which reduced the diffusion of the AFB_1_ molecules. In addition, oil micronutrients, such as vitamin E and sterols, might compete with the AFB_1_ for binding sites on the MCM-41 in the oil phase [10]. Therefore, the adsorption of AFB_1_ was more challenging in an oil-rich matrix such as peanut oil. Nonetheless, MCM-41 showed promising performance in the removal of AFB_1_ in peanut oil and thus had great potential for industrial application.

For elucidating the adsorption process in peanut oil, pseudo-first-order and pseudo-second-order models were used to determine the adsorption kinetics of AFB_1_ onto MONT, MCM-41, and CA. Adsorption kinetics parameters of AFB_1_ obtained with the two kinetic models were presented in Table 2. As shown in Figure 3a,b and Table 2, R^2^ values of the three adsorbents, evaluated in the study obtained with the pseudo-second-order kinetic model, were higher than those obtained with the pseudo-first-order kinetic model. Furthermore, the calculated equilibrium adsorption capacity *q_e_* (cal) provided by the pseudo-second-order model was closer to *q_e_* (exp) than that calculated with the pseudo-first-order model. These results indicated that the pseudo-second-order kinetic model was more suitable in explaining the adsorption kinetics of AFB_1_ by three adsorbents.

### 2.4. Adsorption Isotherm

**Figure 4:**
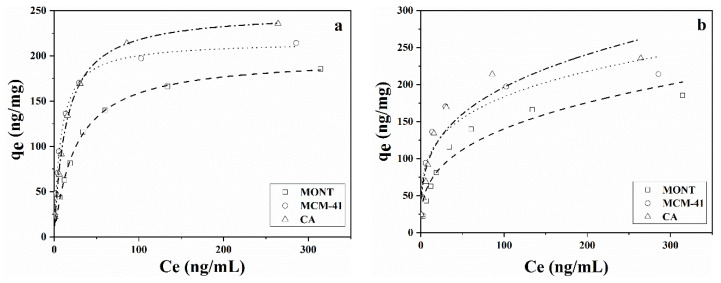
Fit curve of (**a**) Langmuir isotherms and (**b**) Freundlich isotherms of AFB_1_ onto MONT, MCM-41, and CA.

The adsorption isotherms of AFB_1_ on MONT, MCM-41, and CA were evaluated at 25 °C for 24 h with different initial concentrations of AFB_1_ (25–500 ng/mL). Langmuir and Freundlich adsorption isotherms were used to describe the adsorption process of AFB_1_ onto the three adsorbents. Langmuir adsorption isotherm was the most commonly used model describing the monolayer adsorption on a uniform surface and has been successfully applied to describe the adsorption process of many pollutants [48,49]. The Freundlich adsorption isotherm was widely used for empirical formulation to describe adsorption onto a non-uniform surface [44]. Figure 4 depicts the adsorption isotherms of AFB_1_ onto the three adsorbents. The three adsorption isotherms exhibited a quick initial increase and then stabilized, indicating that high-energy adsorption sites were first involved in the adsorption of AFB_1_ at low concentrations. The AFB_1_ adsorption isotherm parameters obtained from the Langmuir and Freundlich models are shown in Table 3. R^2^ values calculated with the Langmuir isotherms were higher than those calculated with the Freundlich isotherms, revealing a better fit of the Langmuir adsorption isotherm in describing the adsorption of AFB_1_. The results also indicated that AFB_1_ was adsorbed in a monomolecular layer onto the surface of the three adsorbents. Moreover, 1/n values from the Freundlich isotherms were below one, indicating a strong interaction between AFB_1_ and the three adsorbents [34]. The monolayer capacities (q_max_) of the three adsorbents calculated based on the Langmuir isotherm were 199.41, 215.93, and 248.93 ng/mg.

## 3. Conclusions

MCM-41 was prepared from rice husk and employed in the removal of AFB_1_ from peanut oil. It showed an adsorption performance comparable to that of commercially available adsorption materials (MONT and CA). The prepared MCM-41 could be considered a good adsorbent candidate for AFB_1_ owing to its high specific surface area, large pore volume, and narrow pore size distribution. The removal rates of AFB_1_ were 73.23%, 80.35%, and 85.09% by MONT, MCM-41, and CA, respectively, when used at a concentration of 1.0 mg/mL. The adsorption kinetics of AFB_1_ in the oil system was lower than those in an aqueous solution, mainly due to the complexity of the oil matrix. The adsorption of AFB_1_ followed quasi-second-order kinetics and fit the Langmuir adsorption isotherm. The calculated maximum adsorbed amounts derived from the Langmuir isotherm were 199.41, 215.93, and 248.93 ng/mg for MONT, MCM-41, and CA, respectively. Collectively, and without modification, MCM-41 had an adsorption performance comparable to that of CA, which was higher than that of MONT. This study demonstrated that MCM-41 may be a suitable adsorption material for the removal of AFB_1_ from peanut oil. This study provided an economic and feasible solution for resourceful utilization of rice husk, which can be applied to effectively reduce AFB_1_ contamination in foods.

## 4. Materials and Methods

### 4.1. Materials and Reagents

Rice husk was supplied from Cofco Engineering Technology Co., Ltd. (Wuxi, China). Peanut oil (viscosity = 10.0 cP; density = 0.915 kg/m^3^ at 20 °C) was purchased from a local supermarket. The standard sample of AFB_1_ (purity ≥ 98%) was purchased from Alexis Corporation, Lausen, Switzerland. Natural montmorillonite (MONT) was purchased from Beijing Enokai Technology Co., Ltd., Beijing, China. Commercial activated carbon (CA) was purchased from ACG Products Ltd., Brookfield, WS, USA. Chromatography grade methanol (purity ≥ 99.9%), toluene (purity ≥ 99.9%), and acetonitrile (purity ≥ 99.9%) were purchased from J&K Scientific, Zhejiang, China. Cetyltrimethylammonium bromide (CTAB, purity ≥ 99%), sodium hydroxide, and hydrochloric acid of analytical grade were purchased from Sinopharm Chemical Reagent Co., Ltd., Shanghai, China.

### 4.2. Synthesis of MCM-41 from Rice Husk

Rice-husk ash was washed with distilled water, diluted in HCl solution (0.1 mol/L), and then dried before use. Washed rice-husk ash (6.4 g) and NaOH (8.0 g) were suspended in 250 mL of distilled water and boiled for 4 h. The mixture was centrifuged (25 °C, 1500× *g*) for 10 min. The supernatant was collected, which was considered a sodium silicate solution, and used as an inorganic silica source. The sodium silicate solution was diluted to a concentration of 5 wt% using distilled water. CTAB (1.0 g) was suspended in 30 mL of 2 mol/L HCl solution. In a typical hydrothermal synthetic procedure, 100 mL of sodium silicate solution and 100 mL of distilled water were added dropwise to 30 mL of the CTAB-HCl solution under vigorous stirring, followed by pH adjusting to 11 using 1 mol/L NaOH solution or 1 mol/L HCl solution. After stirring for 30 min, the mixture was transferred to a hydrothermal reactor for further reaction at 100 °C for 24 h. The precipitated product was centrifuged (25 °C, 3000× *g*) for 10 min, washed with distilled water, and then dried at 80 °C for 12 h. The template was removed by calcination under vacuum at 600 °C at a heating rate of 10 °C/min for 3 h to obtain rice husk-based MCM-41.

### 4.3. Characterization of Rice Husk-Based MCM-41

Nitrogen adsorption–desorption isotherms were measured at 77 K in a JW-BK6 analyzer (Jingwei Gaobo Science and Technology Development Center, Beijing, China). The sample was degassed at 100 °C under vacuum for 24 h prior to measurements. Specific surface areas of the sample were obtained using the Brunauer–Emmett–Teller (BET) method within the relative pressure (p/p_0_) range of 0.05–0.40. The pore size distribution was measured from the adsorption branch using the Barrett–Joyner–Halenda (BJH) method. Total pore volume was calculated from the adsorption capacity at a relative pressure of 0.99. Morphological characterization was performed in a JEOL 2100 transmission electron microscope (JEOL Ltd., Tokyo, Japan) at an accelerating voltage of 200 kV. Powder X-ray diffraction (XRD) was carried out in a Bruker D8 Advance instrument (Bruker Corporation, Karlsruhe, Germany) (40 kV, 200 mA) with Cu Kα radiation (λ = 0.154 nm), and the sample was scanned from 1° to 80° at a rate of 0.02°/s.

### 4.4. Adsorption Experiments

#### 4.4.1. Batch Adsorption Experiments

MONT, MCM-41, and CA at different concentrations (0.1, 0.3, 0.5, 1.0, 1.5, and 3.0 mg/mL) were placed in separate vials containing 10 mL of peanut oil and AFB_1_ (250 ng/mL). Vials were incubated at 25 °C for 24 h under agitation at 150 r/min in an air-shaking platform (HYG-A, Taicang Equipment Factory, Suzhou, China). After adsorption, the supernatant was separated by centrifugation (25 °C, 5000× *g*) for 10 min, and the concentration of AFB_1_ in the supernatant in each vial was determined by HPLC.

#### 4.4.2. Determination of AFB_1_ Concentration

The supernatant (1 mL) containing AFB_1_ was dried with nitrogen at 30 °C, and then 100 μL of trifluoroacetic acid and 200 μL of *n*-hexane were added to the vial. The mixture was instantly stirred for 15 s, derivatized at 40 °C for 30 min, and dried at 30 °C with nitrogen. The residue was dissolved in 1 mL of water–acetonitrile solution (85/15, *v/v*), and the resulting mixture was stirred for 15 s and then centrifuged at 4722× *g* for 5 min. The supernatant was purified by filtration using a 0.22 μm microporous membrane and then transferred to a chromatography vial.

Concentration of AFB_1_ was calculated from chromatograms recorded in an Agilent 1260 series HPLC system (Agilent Technologies, Palo Alto, CA, USA) equipped with a fluorescence detector. Excitation and emission wavelengths were set at 360 and 440 nm, respectively, and an Agilent ZORBAX SB-C18 column was used for separation. A methanol–water binary solvent, at a ratio of 35:65, was used as a mobile phase, and the flow rate was 1.0 mL/min. The removal rate of AFB_1_ was determined using the following equation:

(1) $${\mathrm{AFB}}_{1}\;\mathrm{removal}\;\mathrm{rate}=\left(C_{o}-C_{e}\right)/C_{o}\times 100$$

where *C_o_* is the initial concentration of AFB_1_ (ng/mL) and *C_e_* is the concentration of AFB_1_ in the supernatant at the time of equilibrium (ng/mL).

### 4.5. Adsorption Kinetics

The effect of contact time between AFB_1_ and MONT, MCM-41, and CA was investigated to analyze the adsorption kinetics of AFB_1_. Briefly, 10 mg of each adsorbent was placed in separate vials, and 10 mL of AFB_1_ solution (250 ng/mL) was added to each vial. Adsorption systems were kept at 25 °C under shaking for different periods (10, 30, 60, 120, 150, 180, 240, 480, 960, and 1440 min). The supernatant was separated by centrifugation (25 °C, 5000× *g*) for 10 min and was then used to determine the concentration of AFB_1_ by HPLC at each interval. Pseudo-first-order and pseudo-second-order kinetic models were used to describe the adsorption kinetics of AFB_1_. The equations for the two kinetic models were as follows:

Pseudo-first-order model, *q_t_* = *q_e_*(1 − *e*^−*k*_1_^*^t^*) (2)

(3) $$\mathrm{Pseudo}\text{-}\mathrm{second}\text{-}\mathrm{order}\;\mathrm{model},\;q_{t}=\frac{q_{e}^{2}k_{2}t}{1+k_{2}q_{e}t}$$

where *q_t_* is the amount of AFB_1_ adsorbed on the adsorbent at time *t* (ng/mg), *q_e_* is the amount of AFB_1_ adsorbed on the adsorbent at the time of equilibrium (ng/mg), *k*_1_ is the rate constant of the quasi-first-order model (min^−1^), and *k*_2_ is the rate constant of the quasi-second-order model (mg/(ng·min)). In particular, the equilibrium-adsorption capacity *q_e_* (exp) was measured at an adsorption time equal to 1440 min.

### 4.6. Adsorption Isotherms

The adsorption isotherms were obtained by performing batch equilibrium experiments. Ten milligrams of three adsorbents were added to separate vials and equilibrated with 10 mL of AFB_1_ solution at different initial concentrations (25, 50, 75, 100, 150, 200, 300, and 500 ng/mL) under shaking for 24 h at 25 °C. The concentrations of AFB_1_ in the oil after equilibrium were measured by HPLC. The equilibrium adsorptions were fit by Langmuir and Freundlich isotherm models, and the equations for the two isotherm models are given below.

(4) $$\mathrm{Langmuir}\;\mathrm{isotherm},\;\frac{C_{e}}{q_{e}}=\frac{1}{q_{\max}}C_{e}+\frac{1}{K_{L}q_{\max}}$$

(5) $$\mathrm{Freundlich}\;\mathrm{isotherm},\;1nq_{e}=1nK_{F}+\frac{1}{n}1nC_{e}$$

where *q*_max_ is the saturation capacity of AFB_1_ adsorbed on the adsorbent (ng/mg), *n* is the Freundlich exponent related to surface heterogeneity of the adsorbent, and *K_L_* and *K_F_* are the Langmuir and Freundlich constants, respectively.

### 4.7. Data Analysis

Each experiment was repeated three times. All results were presented as mean ± standard deviation. Data fitting and drawing were performed in Excel 2013 and Origin Pro 9.0. Analysis of variance (ANOVA) was performed in SPSS 17.0 using Duncan’s test to assess differences between sample groups at a confidence level of *p* < 0.05.

## Figures and Tables

**Table 1:** Surface area and pore parameters of MONT, MCM-41, and CA.

Adsorbents	Surface Area (m^2^/g)	Total Pore Volume (cm^3^/g)	Average Pore Diameter (nm)
MONT	235	0.34	1.15
MCM-41	1246	1.75	3.11
CA	453	0.52	1.87

**Table 2:** Adsorption kinetics parameters of AFB_1_ from pseudo-first-order and pseudo-second-order kinetic models.

Adsorbents	q_e_ (exp) (ng/mg)	Pseudo-First-Order Model			Pseudo-Second-Order Model		
		R^2^	q_e_ (cal) (ng/mg)	k_1_ (min^−1^)	R^2^	q_e_ (cal) (ng/mg)	k_2_ (mg/(ng·min))
MONT	192.42	0.9940	166.00	0.00484	0.9960	197.58	2.596 × 10^−5^
MCM-41	217.22	0.9918	188.93	0.00487	0.9981	224.02	2.335 × 10^−5^
CA	229.96	0.9898	199.67	0.00524	0.9974	234.95	2.441 × 10^−5^

**Table 3:** Adsorption isotherm parameters of AFB_1_ calculated by Langmuir and Freundlich adsorption isotherms.

Adsorbents	Langmuir Isotherm			Freundlich Isotherm		
	R^2^	q_max_ (ng/mg)	K_L_	R^2^	K_F_	1/n
MONT	0.9978	199.41	0.0391	0.9106	31.475	0.324
MCM-41	0.9965	215.93	0.128	0.8638	58.807	0.247
CA	0.9949	248.93	0.0701	0.8876	50.889	0.293

## Data Availability

The data presented in this study are available within this article.
